# Cytological Observation and Transcriptome Comparative Analysis of Self-Pollination and Cross-Pollination in *Dendrobium Officinale*

**DOI:** 10.3390/genes12030432

**Published:** 2021-03-17

**Authors:** Yaling Chen, Benchang Hu, Fantao Zhang, Xiangdong Luo, Jiankun Xie

**Affiliations:** College of Life Sciences, Jiangxi Normal University, No 99, Zi Yang Road, Nanchang 330000, China; hubenchang2021@163.com (B.H.); zhang84004@163.com (F.Z.); xdluolf@163.com (X.L.); xiejiankun11@163.com (J.X.)

**Keywords:** *Dendrobium officinale*, cytological observation, transcriptome, self-incompatibility, genes

## Abstract

*Dendrobium officinale* is a rare and traditional medicinal plant with high pharmacological and nutritional value. The self-incompatibility mechanism of *D. officinale* reproductive isolation was formed in the long-term evolution process, but intraspecific hybridization of different germplasm resources leads to a large gap in the yield, quality, and medicinal value of *D. officinale*. To investigate the biological mechanism of self-incompatibility in *D. officinale*, cytological observation and the transcriptome analysis was carried out on the samples of self-pollination and cross-pollination in *D. officinale*. Results for self-pollination showed that the pollen tubes could grow in the style at 2 h, but most of pollen tubes stopped growing at 4 h, while a large number of cross-pollinated pollen tubes grew along the placental space to the base of ovary, indicating that the self-incompatibility of *D. officinale* may be gametophyte self-incompatibility. A total of 63.41 G basesum of *D. officinale* style samples from non-pollinated, self-pollination, and cross-pollination by RNA-seq were obtained, and a total of 1944, 1758, and 475 differentially expressed genes (DEGs) in the comparison of CK (non-pollinated) vs. HF (cross-pollination sample), CK vs. SF (self-pollination sample) and SF vs. HF were identified, respectively. Forty-one candidate genes related to self-incompatibility were found by function annotation of DEGs, including 6 Ca^2+^ signal genes, 4 armed repeat containing (*ARC*) related genes, 11 S-locus receptor kinase (*SRK*) related genes, 2 *Exo70* family genes, 9 ubiquitin related genes, 1 fatty acid related gene, 6 amino acid-related genes, 1 pollen-specific leucine-rich repeat extensin-like protein (*LRX*) related gene and 1 lectin receptor-like kinases (*RLKs*) related gene, showed that self-incompatibility mechanism of *D. officinale* involves the interaction of multiple genes and pathways. The results can provide a basis for the study of the self-incompatibility mechanism of *D. officinale*, and provide ideas for the preservation and utilization of high-quality resources of *D. officinale*.

## 1. Introduction

*Dendrobium officinale* Kimura et Migo, belonging to *Orchidaceae* family, is an ancient and valuable Chinese herbal medicine with high pharmacological and nutritional value, listed as a traditional Chinese herbal in Chinese Pharmacopoeia (State Pharmacopeia Committee of China 2015) [1]. *D. officinale* is mainly distributed in the southern provinces of China, and it is also in East Asia, Southeast Asia, and Australia [2]. The stems and leaves of *D. officinale* contain a variety of effective active ingredients, such as polysaccharides, flavonoids, and alkaloids, which have important pharmacological and edible effects, including the prevention of aging, immunoregulation, antioxidant, and anti-inflammatory effects [3,4,5]. Recently, some studies have found that *D.officinale* also exhibit cure colitis, antiangiogenic, and anti-tumor effects [6,7,8]. The medicinal value of *D. officinale* has gradually been recognized by the public, and wild *D. officinale* has been exploited to near extinction and is now listed as an IUCN (International Union for Conservation of Nature) critically endangered plant.

In the long-term evolution process, the self-incompatibility mechanism of *D. officinale* reproductive isolation has formed, which shows that the pistils and stamens develop normally and mature at the same time, and the seeds can not be produced after self-pollination or cross-pollination with the same genotype [9,10]. Previous studies have shown that the seed setting rate of self-pollination in *D. officinale* was lower than 10%, and that of cross-pollination was higher (80~90%) [11]. The fine varieties of *D. officinale* can only be preserved by intraspecific hybridization or asexual propagation [9]. At present, most of the *D. officinale* in the market are generated through intraspecific hybridization of different germplasm resources, leading to a large gap in the yield, quality, and medicinal value of *D. officinale* [12,13,14]. This further caused confusion in the *D. officinale* market. Therefore, understanding the molecular mechanism of self-incompatibility in *D. officinale* is important for protecting *D. officinale* resources.

Self-incompatibility (SI) is an ancient and common phenomenon which promotes genetic diversity by avoiding self-fertilization in seed plants that possess both stamen and pistil in the same flowers [15]. According to the genetic control modes of pollen SI, it can be divided into two types: sporophytic self-incompatibility (SSI) including *Brassicaceae, Asteraceae and Convolvulaceae* [16], and gametophytic self-incompatibility (GSI) [17], such as *Solanaceae, Scrophyulariaceae, Ranunculaceae, Rosaceae, Onagraceae, Papaveraceae, Leguminosae, and Poaceae* [16]. In GSI, incompatibility depends on the haploid genotype of the pollen, whereas in SSI it depends on the diplotype of its parent [17,18]. GSI is a genetic definition and that pathways in GSI are diverse. In GSI, the S-RNases in the style can degrade the RNA in self pollen tube and the Ca^2+^ mediated pathway are the two most common pathways [19,20]. While the SSI response results from the binding between the S-locus cysteine-rich protein (SCR) in pollen and the S-locus receptor kinase (SRK) that exists on the plasma membrane of the stigma. Thus, its pollen is rejected, completing the SI reaction [21,22,23]. ARC1 (armed repeat containing 1), MLPK (M locus protein kinase), and Exo70A1 (exocyst subunit exo70 family protein A1) are SI-related genes in *Brassica* and *Arabidopsis* [24,25]. Additionally, Ca^2+^ signaling is vital for the process of SSI [26]. Proteomic analyses of pollination in *Dendrobium. chrysanthum* exhibited that β-oxidation of fatty acids, amino acid metabolism, oxidative phosphorylation, and ubiquitin-related protein were associated with SI [27].

*D. officinale* is a perennial epiphytic herbaceous plant of the genus *Dendrobium*. In previous reports, self-incompatibility of *Dendrobium officinale* focused on the observation of seed setting rate and cell biology, but the molecular regulation mechanism is still poorly understood. In the present study, cytological observation was carried out on the samples of self-pollination and cross-pollination in order to identify the SI in *D. officinale*. At the same time, the transcriptome analysis was used to obtain some candidate genes related to SI *D. officinale.* The results can provide a basis for the study of SI mechanism of *D. officinale*, and provide ideas for the preservation and utilization of high-quality resources of *D. officinale*.

## 2. Materials and Methods

### 2.1. Plant Materials and Growth Conditions

Xie et al. [28] indicated that the origin of market individuals were derived from *Shenglan8*, and our previous observation showed that *Shenglan8* and *Longhu1* had high seed setting rate of cross-pollination. So, we selected *Shenglan8* for self-pollination and *Shenglan8* cross-pollination with *Longhu1* to explore cytological and transcriptome comparative phenomena of self-pollination and cross-pollination in *D. officinale.* The tissue-cultured seedlings propagated by stem segments of *D. officinale* were collected from the Jiangxi germplasm resource bank in China. It was cultivated in the laboratory of Jiangxi Normal University. The seedlings were transplanted into pots and cultivated in the growth chamber with a light:dark cycle of 12 h each at 20 to 25 °C and a relative humidity of 60 to 75%; pine bark was used as the substrate. In May 2019, we conducted self-pollination or cross-pollination with sterilized toothpicks on the 3rd to 5th day of flowering. To observe pollen tube growth, nine styles per each sample were gathered at 0 h, 2 h, 4 h, and 8h after self-pollination, respectively, and also gathered at the same time after cross-pollination, then fixed in FAA (formaldehyde:acetic acid:70% alcohol in a ratio of 5:5:90) and stored at −4 °C. For transcriptome sequencing, sixty styles were collected and mixed per each sample that were non-pollinated, self-pollinated (4 h later), and cross-pollinated (4 h later), with three biological duplicates in each group, respectively. The group of non-pollinated samples was labeled as CK (non-pollinated) 1, CK2, CK3; self-pollinated were labeled as SF (self-pollinated)1, SF2, SF3; cross-pollinated were labeled as HF (cross-pollinated) 1, HF2, HF3. All flash-frozen style samples were stored at −80 °C.

### 2.2. Histological Observe of Pollen Tube Growth

The microscopic paraffin-embedded sections were used to observe pollen tube growth to understand the SI of *D. officinale* [29]. The specimens were sliced lengthwise along the stigma using the LEICA microtome RM2016 (LEICA, Shanghai, China). The dyeing procedure was as follows. The first step was dewaxing: paraffin sections were dewaxed by incubating the slices in xylene two times for 20 min each, twice in anhydrous ethanol for 10 min each, followed by incubation in gradient ethanol (95%, 90%, 80%, and 70% alcohol for 5 min each) and finally, distilled water. The second step was staining: the sections were stained with a 1% safranin staining solution for 2 h and rinsed with tap water. The third step was decolorization: the slices were decolorized using 50%, 70%, and 80% gradient alcohol for 1 min each. The fourth step was staining with fast green (FCF) stain: the sections were stained with 0.5% fast green dye solution for 30 to 60s, followed by decolorization in anhydrous ethanol twice for 30 s and 1 min, respectively. The fifth step was the preparation of the cover slice: the slices were baked in a 60 °C oven, kept in xylene solution for 5 min, and finally, the slice was covered with neutral gum. The slices were observed and captured with scanning electron microscope KF-PRO-005 (KFBIO, Ningbo, China).

### 2.3. Total RNA Extraction, Library Preparation and Transcriptome Sequencing

In our study, the styles of non-pollinated, self-pollinated (4 h after pollinated), and cross-pollinated (4 h after pollinated), were ground into powder in mortar with liquid nitrogen, with three biological duplicates in each group, and then the plant rapid extraction kit (Takara, Dalian, China) was used for total RNA extraction from the column samples. The degradation and purity of isolated RNA were studied through 1% agarosegels electrophoresis and IMPLEN NanoPhotometer^®^ spectrophotometer (IMPLEN, California, CA, USA), respectively. Also, the Qubit^®^2.0 Fluorometer (Life Technologies, California, CA, USA) was used for RNA quantification using Qubit^®^ RNA Assay Kit. RNA integrity was tested using the Agilent Bioanalyzer2100 system (Agilent Technologies, California, CA, USA) using the RNA Nano6000 assay kit.

After the RNA sample was qualified using magnetic beads with Oligo (dT), and samples of mRNA enriched by the ways of A-T complementary pairing and binding to the polyA tail of mRNA. Then fragmentation buffer was added to break the mRNA into short fragments. We used mRNA as a template, reverse transcriptase (RNase H-) and a random hexamer primer were used to prepare the first strand of cDNA, followed by the subsequent synthesis of the second strand using RNase H and DNA Polymerase I. Then we used AMPure XP (Beckman Coulter, Beverly, Massachusetts, MA, USA) beads to purify double-stranded cDNA. We performed a purified double-stranded cDNA end repair, added an A tail and connected sequencing adapter, then AMPure XP beads were also used for fragment size selection, and finally, PCR enrichment was performed to obtain the final cDNA library. The cDNA library was sequenced by an Illumina Hiseq2500 platform and 125/150 bp paired-end rawreads were generated. We used in-house perl scripts, and clean reads were generated from the raw reads by removing adapter reads, ploy-N reads, and low-quality reads. Additionally, we calculated Q20, Q30, and GC content of the clean data. Further analyses were done using high-quality clean data.

### 2.4. Reads Mapping to the Reference Genome

The genome database (http://herbalplant.ynau.edu.cn, accessed on 16 March 2021) and the genome database (https://ftp.ncbi.nlm.nih.gov/genomes/all/GCF/001/605/985/GCF_001605985.2_ASM160598v2/GCF_001605985.2_ASM160598v2_genomic.fna.gz, accessed on 16 March 2021) provided the gene model annotation files and the reference genome. Bowtie (v2.2.3) (default parameter) and TopHat (v2.0.12) (Parameter: mismatch = 2) were utilized to build the reference genome index and to align the clean reads to the reference genome, respectively. TopHat was chosen since it was superior to contemporary tools as it could generate a gene model annotation-based splice junction database.

### 2.5. Identification of Differentially Expressed Genes (DEGs)

Gene expression was calculated as reads per kilobase of transcript, per million reads (RPKM). The fragment length and corresponding mapped reads were used to calculate FPKM using Cuffquant and cuffnorm (v2.2.1) in each sample [30]. Here, the summation of FPKMs of transcripts in each gene group for DESeq2 provided the gene expression levels [31]. DESeq2 R package (v 1.10.1) was used to conduct differential expression analysis of two conditions/groups (two biological duplicates/conditions). DESeq2 facilitated the determination of differential gene expression using a negative binomial distribution-based model. The resulting *p*-values were adjusted to account for the FDR, and those with the adjusted *p*-value < 0.01 were classified as DEGs.

### 2.6. Function Annotation and Enrichment Analysis

In order to know the function of unigene, we used databases including Nr (ftp://ftp.ncbi.nlm.nih.gov/blast/db, accessed on 16 March 2021), Swiss-Prot (http://ftp.ebi.ac.uk/pub/databases/swissprot/, accessed on 16 March 2021), TrEMBL (https://www.uniprot.org/news/2004/03/02/full, accessed on 16 March 2021), KOG (ftp://ftp.ncbi.nih.gov/pub/COG/KOG, accessed on 16 March 2021), Pfam (http://pfam.xfam.org/, accessed on 16 March 2021), GO (http://geneontology.org/, accessed on 16 March 2021), COG (https://www.ncbi.nlm.nih.gov/COG, accessed on 16 March 2021), and KEGG (http://www.genome.jp/kegg/, accessed on 16 March 2021) to annotate the unigene of transcriptome sequencing and the DEGS. To find the DEGs associated with SI, GO, COG, and KEGG databases were used to function enrich and classify the DEGS of SF vs. HF. The Gene Ontology (GO) seq R package was utilized for GO enrichment analysis of DEGs, which involved the correction of the gene length bias, and those with corrected *p*-value < 0.05 were regarded as significantly enriched in DEGs. The KEGG database was used for understanding the protein network analysis (http://www.genome.jp/kegg/, accessed on 16 March 2021). We studied the DEGs enriched in KEGG pathways via the KOBAS software (v2.0, Beijing, China).

### 2.7. qRT-PCR Validate Analysis

In this study, we used qRT-PCR to analyze DEGS levels in SF vs. HF to verify the reliability of transcriptome sequencing data. The PrimeScript^TM^ RT reagent kit (Takara, Dalian, China) with gDNA Eraser was used for cDNA synthesis. Primer 3.0 (v0.4.0) was utilized for designing the gene primers sequences for qRT-PCR analysis (Supplementary Table S1). The StepOne^TM^ Real-Time PCR System (ABI, New York, NY, USA) was used to perform qRT-PCR using the 2×Q3 SYBR qPCR Master mix (TOLOBIO, Shanghai, China). Each experiment was performed in triplicates. The gene expression levels were normalized using the *GAPDH* gene [32]. Relative quantification of genes expression was calculated based on the 2^−ΔΔCT^ method [33].

## 3. Results

### 3.1. Observation of Pollen Tube Growth

**Figure 1:**
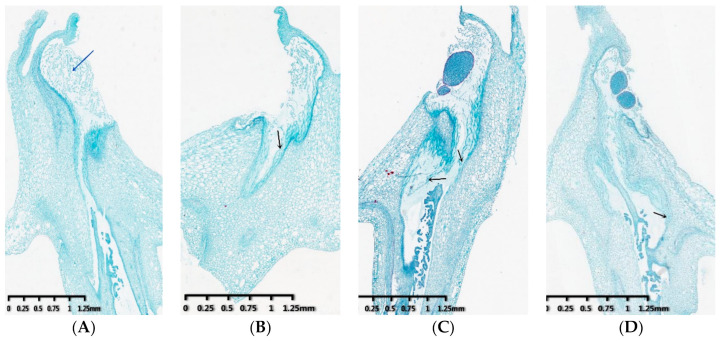

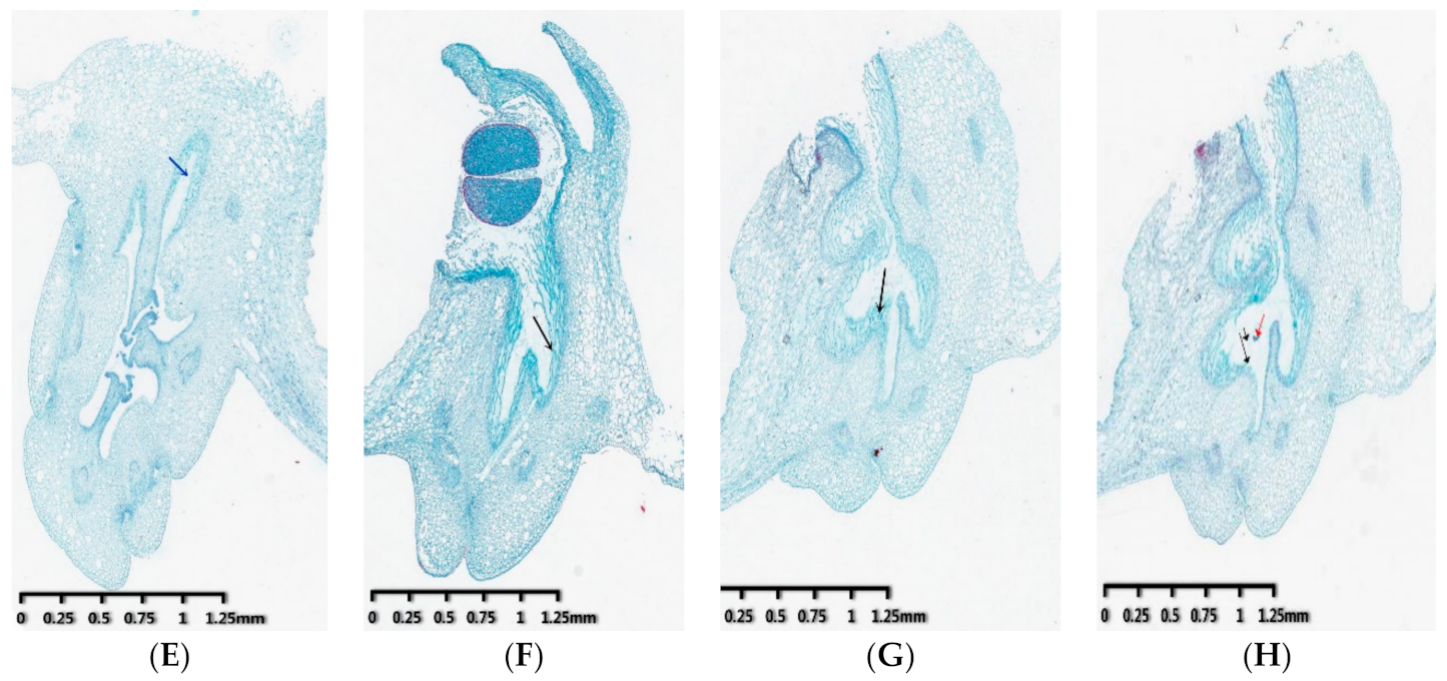
The growth of pollen tube after self-pollination and cross-pollination in the pistils. (**A**) The picture of non-pollination in self-pollination. (**B**) The growth of pollen tube in 2 h after self-pollination. (**C**) The growth of pollen tube 4 h in after self-pollination. (**D**) The growth of pollen tube 8 h in after self-pollination. (**E**) The picture of non-pollination in cross-pollination. (**F**) The growth of pollen tube in 2 h after cross-pollination. (**G**) The growth of pollen tube in 4 h after cross-pollination. (**H**) The growth of pollen tube in 8 h after cross-pollination. The blue arrow point to the stigma, the black arrow point to the pollen tube, and the red arrow point to the ovule.

The growth of pollen tubes in the style at 0 h, 2 h, 4 h, 8 h after self-pollination and cross-pollination was observed by scanning electron microscope, respectively. Results showed that there are guiding tissues on the pistil stigma (blue arrow), and pollen tubes (black arrow) in the style are clustered together and grow longitudinally along the two walls at 0 h after self-pollination and cross-pollination (Figure 1A,E). At 2 h of pollination, the self-pollinated pollen tube grows to the middle of the style, while the cross-pollinated pollen tube grows to the style bottom, and the pollen tubes of cross-pollination are obviously more than those of self-pollination (Figure 1B,F). At 4 h of pollination, most of the self-pollinated pollen tubes stopped growing and only a few slowly grew to the style bottom (Figure 1C). Although some cross-pollinated pollen tube growth was arrested in the styles, lots of pollen tubes still grew to the style bottom at 4 h of pollination (Figure 1G). At 8 h of self-pollination, the pollen tubes that grew to the upper part of the style had disappeared and a few pollen tubes kept growing slowly to the bottom of the style (Figure 1D), while the pollen tubes continue to grow towards the bottom of the ovary, some of them have reached the ovule (red arrow) after cross-pollination (Figure 1H). These observations indicate that the growth of pollen tubes in the style is significantly inhibited after self-pollination in *D. officinale.*

### 3.2. Transcriptome Sequencing Analysis

In the present study, non-pollinated, self-pollinated (4 h later), and cross-pollinated (4 h later) column samples were collected to perform RNA-seq analysis. Three groups of cDNA libraries (non-pollinated: CK1, CK2, CK3; self-pollination: SF1, SF2, SF3; cross-pollination: HF1, HF2, HF3) were separately prepared. In this study, the raw data contained 63,409,511,100 baseSum. After removing the low-quality sequences, we obtained 211,365,037 readSum. The average Q20, Q30 values, and GC content was 97.56%, 93.24%, and 47.63%, respectively (Table 1). Thus, the data of the transcriptome sequencing had high stability. In addition, 71.58% of the clean reads were mapped to the reference genome (Table 2). Through this result, it can be evaluated that the selected reference genome assembly can meet the needs of information analysis.

Previous studies have clarified that there are biological differences in gene expression among different individuals [34]. Thus, for studies involving biological duplicates, it is necessary to assess their relevance to analyze transcriptome sequencing data. Here, we used Pearson’s correlation coefficient (R^2^) as an indicator of biological repeat correlation [35]. All R^2^ values were >0.78 amongst different biological duplicates and were >0.55 between different samples (Supplementary Figure S1). In addition, we analyzed all of samples gene expression clustering and we found insignificant differences among the duplicated samples and groups from the heat map (Supplementary Figure S2).

The unigenes in three transcriptome sequencing databases were annotated in the databases (a total of 38,373 unigenes). These unigenes were annotated in public databases including Nr, Swiss-Prot, TrEMBL, Pfam, KOG, GO, COG, and KEGG databases. Among them, there were 38,343 unigenes in Nr database, accounting for 99.92%, 35,450 unigenes in TrEMBL database, accounting for 92.38% and 27,694 comments in Swiss-Prot database, accounting for 72.17%, respectively. A total of 26,003 unigenes annotated in Ptam databases, accounting for 67.76%. The numbers of unigenes in KOG databases 22,346 (accounting for 58.28%). Besides, 15,993 unigenes (accounting for 41.68%) annotated in GO databases, 10,665 unigenes (accounting for 27.80%) annotated in COG database, 6775 unigenes (accounting for 17.66%) annotated in KEGG databases, respectively (Supplementary Figure S3), showed that many metabolic pathways are involved in self- and cross-pollinated of *D. officinale*.

### 3.3. Identification of Differentially Expressed Genes (DEGs)

**Figure 2:**
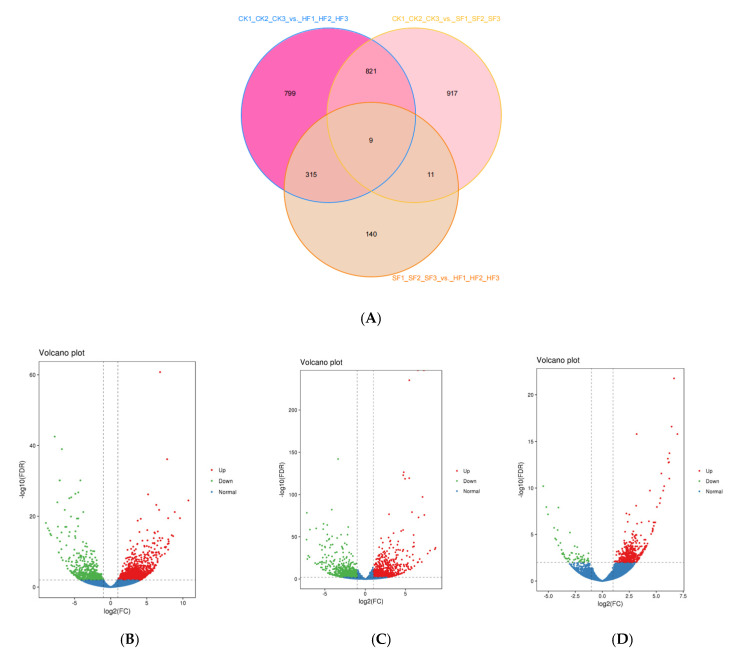
Statistics of genes with differential expressions in the CK1, CK2, CK3; HF1, HF2, HF3; SF1, SF2, SF3 libraries. (**A**) Venn diagrams of the unique and common DEGs among the three libraries. (**B**–**D**) Volcano plot of the identified DEGs in the comparisons of CK vs. HF, CK vs. SF, and SF vs. HF.

We used FDR (false discovery rate) <0.01 and log_2_FC (fold Change) ≥2 as the selection parameters to identify DEGs. The fold Change implied the expression ratio between the two sample groups. The FDR was achieved by correcting the difference for significant *p*-values. Here, the comparison of CK1_CK2_CK3_vs._HF1_HF2_HF3, CK1_CK2_CK3_vs._SF1_SF2_SF3, and SF1_SF2_SF3_vs._HF1_HF2_HF3 yielded 1944, 1758, and 475 DEGs, respectively (Table 3 and Supplementary Figure S4). Of these, nine genes were commonly expressed in the CK, SF, and HF libraries based on the Venn diagram (Figure 2A). Amongst the detected DEGs, the CK1_CK2_CK3_vs_HF1_HF2_HF3 group had 1358 upregulated and 586 downregulated genes (Figure 2B), the CK1_CK2_CK3_vs._SF1_SF2_SF3 group had 932 up-regulated and 826 down-regulated genes (Figure 2C), and the SF1_SF2_SF3_ vs._HF1_HF2_HF3 group had 417 up-regulated and 58 down-regulated genes (Figure 2D). Particularly, the SF1_SF2_SF3_ vs. _HF1_HF2_HF3 group had fewer DEGs than the other two groups.

### 3.4. Functional Annotation and Enrichment of DEGS

**Figure 3:**
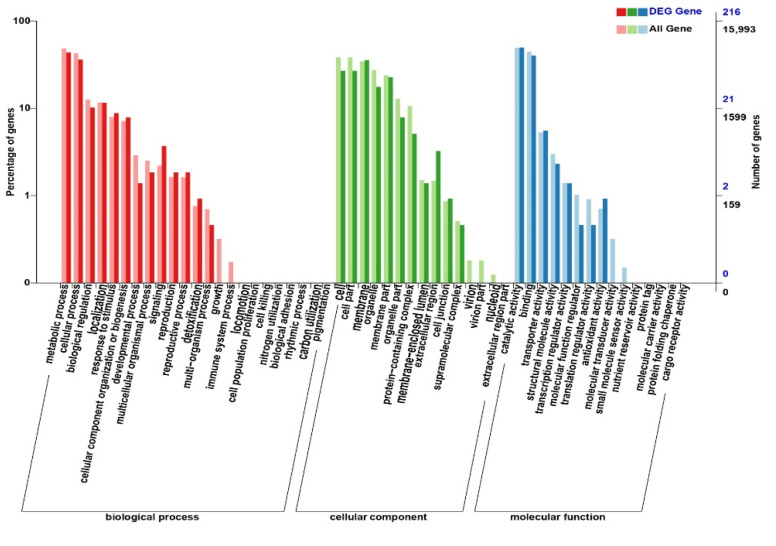
The GO terms classification of all gene and DEGS in SF vs. HF. The annotated genes are divided into three functional GO categories: biological process (BP), cellular component (CC), and molecular function (MF).

In this study, we annotated the DEGs in the SF vs. HF group using Swiss-Prot, Nr, Pfam, KOG, GO, KEGG, and COG databases to find SI-related genes. To explore the role of DEGs, we conducted the GO term classification analysis based on sequence homology and COG function (Figure 3, Table 4), and KEGG pathway enrichment analysis of the DEGs in the SF vs. HF group. DEGs were functionally classified to understand their biological functions.

**Figure 4:**
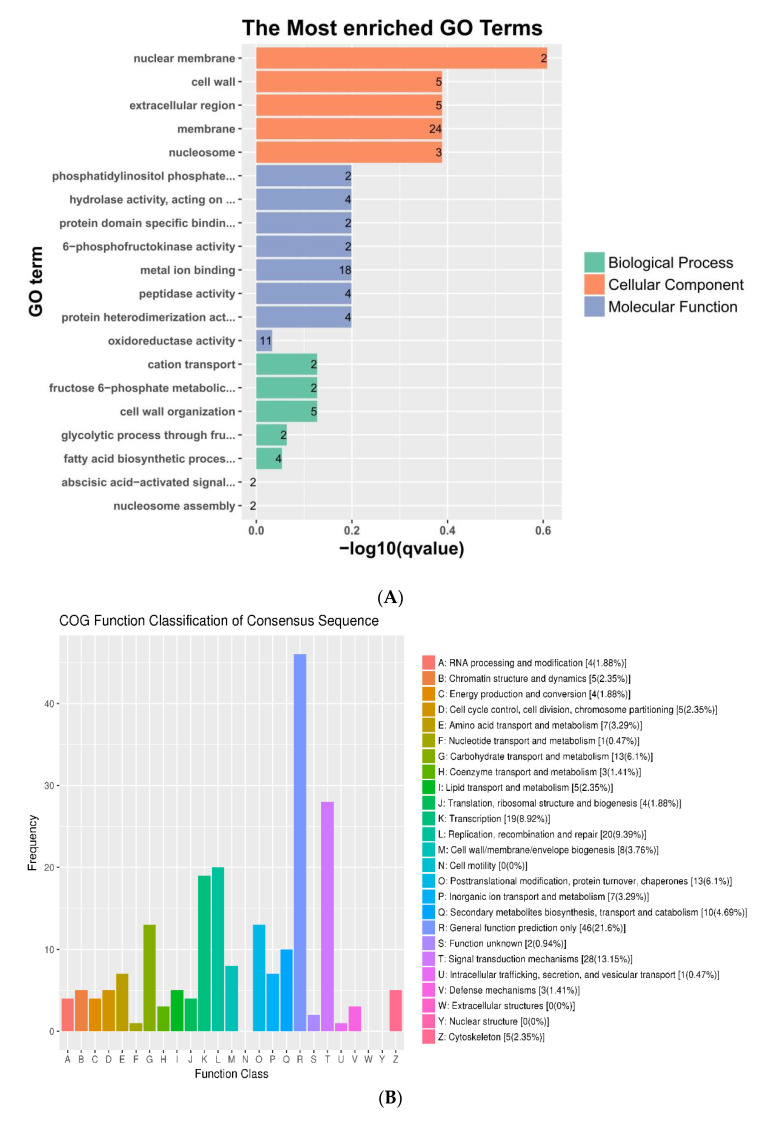

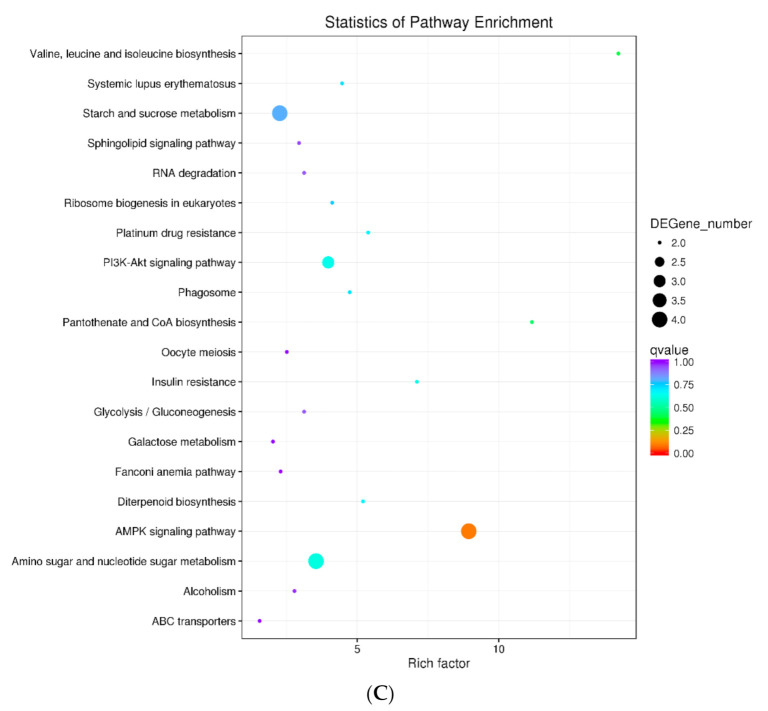
Analysis of functional classification and enrichment of DEGS in SF vs. HF. (**A**) The top 20 most enriched GO terms of DEGS in SF vs. HF. (**B**) The COG function classification of DEGS in SF vs. HF. (**C**) The top 20 most enriched KEGG pathways of DEGS in SF vs. HF. PI3K (Phosphatidylinositol-3-kinase), AKT (Silk/threonine protein kinase), COA (coenzyme A), AMPK (Adenosine Monophosphate Activated Protein Kinase), ABC (ATP-binding cassette).

Here, we found that all DEGs were grouped into 53 functional terms in GO terms, and could be classified into the following functional GO categories: cellular component (CC), biological process (BP), and molecular function (MF). For the groups CK vs. SF and CK vs. HF, 813 and 815 DEGs were assigned to the GO terms, respectively (Supplementary Figure S5A,B). However, in the group SF vs. HF, only 216 DEGs were assigned to the GO term (Figure 3). In all three groups, the two most abundant terms “metabolic process (48.35%)” and “catalytic activity (49.25%)” were identical. For CC, these terms were “cell (38.31%)” and “cell part (38.24%)”. For BP, the two most abundant terms were “metabolic process (48.35%)” and “cellular process (42.72%)” included cation transport, cell wall organization, glycolytic process through fructose-6-phosphate, fatty acid biosynthetic process, fructose 6-phosphate metabolic process, abscisic acid-activated signaling pathway, and nucleosome assembly (Figure 4A). For MF, the terms were “catalytic activity (49.25%)” and “binding (44.33%)” (Figure 3) included phosphatidylinositol phosphate kinase activity, hydrolase activity, esterolysis, protein domain specific binding, 6-phosphofructokinase activity, metal ion binding, peptidase activity, protein heterodimerization activity, and oxidoreductase activity (Figure 4A). This study speculated that after self-pollination, various metabolic pathways and biological regulatory processes in *D. officinale* were affected to varying degrees, which further affected the expression of mRNA and target genes regulated by mRNA (Table 4).

In COG database, 213 differentially expressed genes were successfully annotated and classifified into 22 function classes. Among them, sequences involved in general function prediction only (21.6%); signal transduction mechanisms (13.15%); replication, recombination and repair (9.39%); transcription (8.92%); carbohydrate transport and metabolism and posttranslation modiication, protein trun over, chaperones were highly expressed (Figure 4B).

Moreover, KEGG pathway enrichment of the DEGS was also analyzed in this study. In the KEGG database, 50 differentially expressed genes were annotated and enriched into 98 pathways. Among them, a total of 33 pathways were obviously enriched. Figure 4C shows that the top 20 most enriched pathways, including valine, leucine, and isoleucine biosynthesis; systemic lupus erythematosus; starch and sucrose metabolism; sphingolipid signaling pathway and RNA degradation and so on (Figure 4C).

In the above three function enrichment, some particular pathways were observed, such as cation transport, metal ion binding, RNA degradation, and signal transduction mechanisms. Additionally, fatty acid biosynthetic process, transcription, and unknown pathway were identified. These pathways may be associated with self-incompatibility. So it is necessary to conduct further research on the genes related to these pathways and find candidate genes related to self-incompatibility.

### 3.5. Identification of Candidate Genes Associated with Self-Incompatibility

We selected some SI-related candidate genes from *D. officinale* based on the function and protein of annotated DEGs identified in the SF vs. HF group. Forty-one SI-related candidate genes were identified from DEGs (Table 5). Among these, six calcium ion sensor proteins were identified, including four calmodulin-like protein (CML), one calcium-dependent protein kinase (CPK), and one calcineurin B-like protein (CBL). For instance, DoLRX4 protein was found to facilitate rapid pollen tube wall growth. Lectin receptor-like kinases (Lectin RLKs) possess an extracellular lectin-like domain. Additionally, four ARC-related genes, eleven SRK-related genes, two Exo70 family genes, nine ubiquitin-related genes, one fatty acid-related genes, and six amino-acid-related genes were identified (Table 5).

### 3.6. qRT-PCR Validate Analysis of DEGs

**Figure 5:**
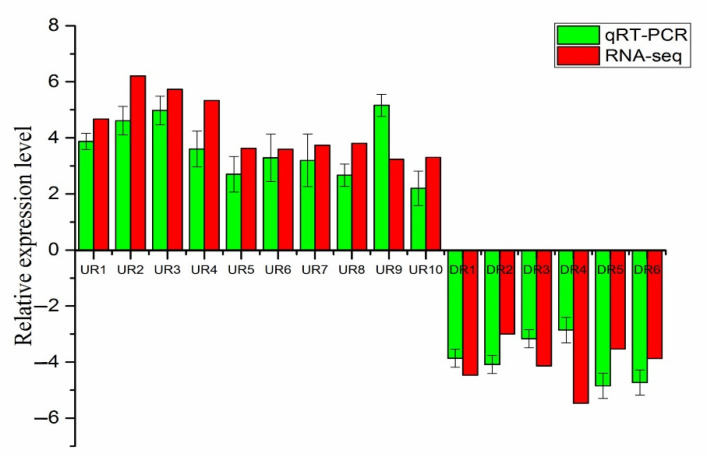
Validation of RNA-seq data by qRT-PCR analysis. X-axis: gene name, Y-axis: the relative expression level was expressed as log_2_(fold change) in gene expression. The relative expression of 16 random genes, were determined by RT-qPCR (green column) and compared with the results of RNA-seq (red column). Error bars represented standard deviation (SD). UR (up-regulated genes), DR (down-regulated genes).

We randomly selected 16 DEGs (10 up-regulated and 6 down-regulated) in the SF vs. HF group for qRT-PCR analysis to evaluate the precision of transcriptome sequencing. The comparative expression of SF and HF was used to compare between RNA-seq and qRT-PCR. Here, the regulatory trends of 16 selected DEGs in RNA-seq and qRT-PCR were roughly the same (Figure 5). The qRT-PCR result indicated that the DEGs of transcriptome sequencing data were reliable.

## 4. Discussion

Previous research showed that gametophyte self-incompatibility indicated that pollen could invade the stigma after germination on the stigma, and could extend for a section in the style tissue, and then was inhibited. However, sporophytic self-incompatibility indicated that pollen falls on the stigma and fails to germinate normally, or it wraps around the stigma papillary cells after germination but cannot invade the stigma [41,42]. In this study, the microscopic observation showed that the pollen tubes could grow in the style at 2h after self-pollination and cross-pollination, but most of the pollen tubes stopped growing at 4h after self-pollination and some pollen tubes had disappeared at 8h after self-pollination (Figure 1), consistent with results of previous studies [43]. Studied on pollen tube germination in *D. officinale* showed that SI response after self-pollination initiated in a short time with less than 4h. The results indicated that self-incompatibility of *D. officinale* may be gametophyte self-incompatibility, while the specific SI types still need to be verified by molecular biological evidence.

The studies on SI of *Citrus reticulata* had shown that SI-related candidate genes acted by regulating pollen development, the ubiquitin pathway, signaling pathways, gibberellin stimulus, receptor kinases, calcium ion binding, and transcription [44,45]. Here, we detected that cation transport, metal ion binding, RNA degradation, and signal transduction mechanisms (Figure 4) were associated with SI. The expression of Lectin RLKs was up-regulated in the HF group (Table 5). Wan et al. (2008) [40] showed that male sterility in *Arabidopsis* was due to mutation in one Lectin RLK gene. So Lectin RLKs may play an important role in cross-pollination of *D. officinale. DoLRX4* levels that were also up-regulated in HF (Table 5). *DoLRX4* similar to *LRX(ZmPEX1*) was specifically expressed in *Zea Mays* pollen. *ZmPEX1* is known to be located in the inner wall of pollen grains and callose sheath of the pollen tube. Studies have substantiated the role of *ZmPEX1* in reproduction, which implies that it may be involved in the precipitation of structural units during the rapid growth of the pollen tube wall, or it may act as a sex recognition signal molecule interacting with the pistillate [39].

Calcium ion sensor proteins, an important class of proteins, are involved in several signaling pathways in plants [46]. Here, six calcium ion sensor proteins, including four CML protein, one CPK, and one CBL were identified from DEGs, and these proteins have been previously identified and found to be related to SI in *Brassica* [36,37,38,47]. Additionally, the expression of some *CaM/CML* (Calmodulin/calmodulin-like) genes has been demonstrated in *A. thaliana* during pollen germination and tube growth [48]. Thus, calcium ion sensor proteins, may play an important regulatory role in the SI of *D. officinale*.

Here, four ARC-related genes, eleven SRK, and two Exo70 family genes were identified from DEGs (SF vs. HF). ARC1 is known to bind to activated SRK, followed by phosphorylation, which results in the degradation of EXO70A1 [20,21]. Ubiquitin is a proteolytic degradation marker and it is hypothesized that the activation of SI responses might be dependent on a phosphorylation-mediated ubiquitination mechanism [49,50]. Also, the ubiquitination mechanism is the key regulator of the S-RNase-based GSI [51]. Besides, we also found some genes for amino acid metabolism (Figure 4, Table 5). The proteins produced by these genes act as critical enzymes that convert glutamate to glutamine, which facilitates the assimilation of ammonia into glutamine for its transport in plants [52,53]. Additionally, glutamate is a γ-aminobutyrate precursor, which regulates pollen tube growth [54].

In conclusion, some genes that may play a role in SI of *D. officinale* have been discovered in our research, including Ca^2+^ signal genes, *ARC* related genes, *SRK* related genes, *Exo70* family genes, ubiquitin-related genes, fatty acid-related genes, amino acid-related genes, *LRX*-related genes and *RLKs*-related genes. This result provides some candidate genes to research the specific mechanism of SI in *D. officinale.*

## Figures and Tables

**Table 1:** The information of the sequenced transcriptome in *Dendrobium officinale.*

#Sample ID	ReadSum	BaseSum	GC(%)	Q20(%)	Q30(%)
CK1	22,450,889	6,735,266,700	47.49%	97.83%	93.76%
CK2	21,276,069	6,382,820,700	47.25%	97.14%	92.14%
CK3	24,726,730	7,418,019,000	47.09%	97.46%	92.97%
CK average	22,817,896	6,845,368,800	47.28%	97.48%	92.96%
HF1	28,969,506	8,690,851,800	52.62%	97.29%	92.85%
HF2	20,949,503	6,284,850,900	47.06%	97.64%	93.40%
HF3	21,063,503	6,319,050,900	46.80%	97.74%	93.63%
HF average	23,660,837	7,098,251,200	48.83%	97.56%	93.29%
SF1	20,991,442	6,297,432,600	46.87%	97.71%	93.55%
SF2	27,714,690	8,314,407,000	46.88%	97.60%	93.33%
SF3	23,222,705	6,966,811,500	46.65%	97.65%	93.49%
SF average	23,976,279	7,192,883,700	46.80%	97.65%	93.46%
Total average	23,485,004	7,045,501,233	47.63%	97.56%	93.24%
Total	211,365,037	63,409,511,100			

Sample ID: sample name; ReadSum: clean data total number of pair-end Reads; BaseSum: clean data total base number; GC (%): clean data GC content, clean data G and C bases account for total alkali percentage of base; Q20 (%): a sequencing error probability of 1%; Q30 (%): a sequencing error probability of 0.1%.

**Table 2:** The table of the sequence alignment between sequencing data and the reference genome.

Sample ID	Total Read	Reads Mapped
CK1	44,901,778	33,273,053 (74.10%)
CK2	42,552,138	31,039,066 (72.94%)
CK3	49,453,460	34,715,952 (70.20%)
HF1	57,939,012	39,336,622 (67.89%)
HF2	41,899,006	29,965,533 (71.52%)
HF3	42,127,006	30,322,911 (71.98%)
SF1	41,982,884	30,649,680 (73.01%)
SF2	55,429,380	40,043,108 (72.24%)
SF3	46,445,410	33,240,722 (71.57%)
Total	422,730,074	302,586,647 (71.58%)

Sample ID: Sample name; Total read: The number of Clean Reads is counted as two Reads in one pair; Reads mapped: The number of Reads mapped to the reference genome and the percentage of Reads in Clean Reads.

**Table 3:** The number of differential expression gene in *Dendrobium officinale*.

DEG Set	All DEG	Up-Regulated	Down-Regulated
CK1_CK2_CK3_ vs._HF1_HF2_HF3	1944	1358	586
CK1_CK2_CK3_ vs._SF1_SF2_SF3	1758	932	826
SF1_SF2_SF3_ vs._HF1_HF2_HF3	475	417	58

DEG Set: differential expression gene set name; All DEG: number of differentially expressed genes; up-regulated: number of up-regulated genes; down-regulated: number of down-regulated genes.

**Table 4:** The number of total genes and number of DEGS of GO term in SF vs. HF.

GO Classify	All Gene	DEG Gene	Ratio
extracellular region	238	7	0.0294
signaling	352	8	0.0227
antioxidant activity	113	2	0.0177
detoxification	122	2	0.0164
reproductive process	259	4	0.0154
reproduction	261	4	0.0153
response to stimulus	1275	19	0.0149
cellular component organization or biogenesis	1146	17	0.0148
cell junction	138	2	0.0145
transporter activity	845	12	0.0142
membrane	5497	77	0.0140
catalytic activity	7876	107	0.0136
Total gene	15,993	216	0.0135
transcription regulator activity	224	3	0.0134
localization	1869	25	0.0134
membrane part	3812	49	0.0129
membrane-enclosed lumen	240	3	0.0125
binding	7089	87	0.0123
supramolecular complex	82	1	0.0122
metabolic process	7732	94	0.0122
cellular process	6833	78	0.0114
biological regulation	2010	22	0.0109
structural molecule activity	478	5	0.0105
multicellular organismal process	404	4	0.0099
cell part	6115	58	0.0095
cell	6127	58	0.0095
multi-organism process	112	1	0.0089
organelle	4386	38	0.0087
organelle part	2062	17	0.0082
translation regulator activity	146	1	0.0068
protein-containing complex	1693	11	0.0065
developmental process	466	3	0.0064
molecular function regulator	163	1	0.0061
nucleoid	20	0	0
virion	29	0	0
extracellular region part	11	0	0
virion part	29	0	0
protein tag	3	0	0
cargo receptor activity	1	0	0
protein folding chaperone	2	0	0
nutrient reservoir activity	9	0	0
molecular transducer activity	51	0	0
molecular carrier activity	3	0	0
small molecule sensor activity	24	0	0
cell killing	4	0	0
immune system process	28	0	0
cell population proliferation	5	0	0
carbon utilization	2	0	0
nitrogen utilization	4	0	0
biological adhesion	3	0	0
growth	51	0	0
locomotion	10	0	0
pigmentation	2	0	0
rhythmic process	3	0	0

**Table 5:** The candidate genes associated with self-incompatibility from DEGS in SF vs. HF.

Gene Name	ID	Description	Regulated (log_2_FC)	References
*DoCML11*	*Dendrobium_GLEAN_10039171*	Calmodulin-like protein 11	Up (3.59)	[36]
*DoCML14*	*Dendrobium_GLEAN_10057311*	Putative calcium-binding protein CML14	Up (2.77)	[36]
*DoCML10*	*Dendrobium_GLEAN_10061210*	Probable calcium-binding protein CML10	Up (2.61)	[36]
*DoCML42*	*Dendrobium_GLEAN_10078224*	Calcium-binding protein CML42	Up (2.55)	[36]
*DoCPK20*	*Dendrobium_GLEAN_10069252*	Calcium-dependent protein kinase 20	Up (2.17)	[37]
*DoCBL7*	*Dendrobium_GLEAN_10021981*	Calcineurin B-like protein 7	Down (−1.67)	[38]
*DoLRX4*	*Dendrobium_GLEAN_10140109*	Pollen-specific leucine-rich repeat extensin-like protein 4	Up (2.81)	[39]
*DoL-RLKs1*	*Dendrobium_GLEAN_10110992*	L-type lectin-domain containing receptor kinase IX.1	Up (2.29)	[40]
*DoARC12*	*Dendrobium_GLEAN_10051179*	U-box domain-containing protein 12	Up (2.35)	[23]
*DoARC52*	*Dendrobium_GLEAN_10116227*	U-box domain-containing protein 52	Up (2.25)	[23]
*DoARC15*	*Dendrobium_GLEAN_10032857*	U-box domain-containing protein 15	Up (2.18)	[23]
*DoARC3*	*Dendrobium_GLEAN_10017335*	U-box domain-containing protein 3	Up (2.25)	[23]
*DoSRK1*	*Dendrobium_GLEAN_10067876*	Putative inactive leucine-rich repeat receptor-like protein kinase	Up (2.68)	[21,22]
*DoSRK2*	*Dendrobium_GLEAN_10005387*	CBL-interacting serine/threonine-protein kinase 12	Up (2.43)	[21,22]
*DoSRK3*	*Dendrobium_GLEAN_10113311*	serine/threonine-protein kinase D6PKL2	Up (2.78)	[21,22]
*DoSRK4*	*Dendrobium_GLEAN_10042125*	CBL-interacting serine/threonine-protein kinase 12	Up (2.62)	[21,22]
*DoSRK5*	*Dendrobium_GLEAN_10128039*	serine/threonine-protein kinase BLUS1	Up (2.13)	[21,22]
*DoSRK6*	*Dendrobium_GLEAN_10027995*	Putative serine/threonine-protein kinase	Up (2.03)	[21,22]
*DoSRK7*	*Dendrobium_GLEAN_10098521*	Serine/threonine-protein kinase PBS1	Up (1.86)	[21,22]
*DoSRK8*	*Dendrobium_GLEAN_10000384*	CBL-interacting serine/threonine-protein kinase 12	Up (2.43)	[21,22]
*DoSRK9*	*Dendrobium_GLEAN_10010756*	Leucine-rich repeat receptor-like serine/threonine-protein kinase	Up (2.06)	[21,22]
*DoSRK10*	*Dendrobium_GLEAN_10047407*	Receptor-like serine/threonine-protein kinase ALE2	Down (−1.32)	[21,22]
*DoSRK11*	*Dendrobium_GLEAN_10044962*	Putative LRR receptor-like serine/threonine-protein kinase RLK	Up (2.41)	[21,22]
*DoEXO1*	*Dendrobium_GLEAN_10047257*	Exocyst complex component EXO70B1	Up (2.68)	[24,25]
*DoEXO2*	*Dendrobium_GLEAN_10108122*	Exocyst complex component EXO70B1	Up (2.83)	[24,25]
*DoEBI1*	*Dendrobium_GLEAN_10013195*	E3 ubiquitin-protein ligase WAV3	Up (2.24)	[27]
*DoEBI2*	*Dendrobium_GLEAN_10030613*	E3 ubiquitin-protein ligase RGLG4	Up (2.13)	[27]
*DoEBI3*	*Dendrobium_GLEAN_10106149*	E3 ubiquitin-protein ligase SPL2	Up (1.62)	[27]
*DoEBI4*	*Dendrobium_GLEAN_10029117*	E3 ubiquitin-protein ligase KEG	Up (2.49)	[27]
*DoEBI5*	*Dendrobium_GLEAN_10062294*	Ubiquitin-like protein-NEDD8-like protein RUB3	Up (3.09)	[27]
*DoEBI6*	*Dendrobium_GLEAN_10032857*	Ring-type E3 ubiquitin transferase	Up (2.18)	[27]
*DoEBI7*	*Dendrobium_GLEAN_10126475*	E3 ubiquitin-protein ligase XBAT31	Up (2.07)	[27]
*DoEBI8*	*Dendrobium_GLEAN_10076237*	Probable BOI-related E3 ubiquitin-protein ligase 3	Up (2.47)	[27]
*DoEBI9*	*Dendrobium_GLEAN_10069672*	BOI-related E3 ubiquitin-protein ligase 1	Up (2.43)	[27]
*DoFAT1*	*Dendrobium_GLEAN_10055554*	omega-6 fatty acid desaturase	Up (2.34)	[27]
*DoAMI1*	*Dendrobium_GLEAN_10138041*	Serine carboxypeptidase-like 35	Up (2.31)	[27]
*DoAMI2*	*Dendrobium_GLEAN_10118990*	Amino-acid permease BAT1 like	Down (−2.86)	[27]
*DoAMI3*	*Dendrobium_GLEAN_10033197*	Aspartokinase 1	Up (1.57)	[27]
*DoAMI4*	*Dendrobium_GLEAN_10141386*	Arogenate dehydratase/prephenate dehydratase 2	Up (2.21)	[27]
*DoAMI5*	*Dendrobium_GLEAN_10014762*	Probable polyamine transporter At3g19553	Up (3.17)	[27]
*DoAMI6*	*Dendrobium_GLEAN_10056253*	amino acid transporter	Up (1.53)	[27]

## Supplementary Materials

The following are available online at https://www.mdpi.com/2073-4425/12/3/432/s1, Table S1.: The Sequences of primer for qRT-PCR validate analysis; Figure S1.: Pearson correlation of all samples; Figure S2.: Heat map of gene expressions in the three libraries with three replicates; Figure S3.: The number of the unigenes annotated in the databases; Figure S4. The number of the identified DEGs in the three libraries; Figure S5.: The GO terms classification of all gene and DEGS. (**A**) GO classification annotation in CK1_CK2_CK3_ vs. _SF1_SF2_SF3. (**B**) GO classification annotation in CK1_CK2_CK3_ vs. _HF1_HF2_HF3.
