# Supplementary material for: Cytological Observation and Transcriptome Comparative Analysis of Self-Pollination and Cross-Pollination in Dendrobium Officinale
Source: Genes (Basel). 2021 Mar 17;12(3):432. doi: 10.3390/genes12030432 (PMC8002659; doi:10.3390/genes12030432)
Supplement: Supplementary file 1 [file genes-12-00432-s001.pdf]

Supplementary Table

Table S1. The Sequences of primer for qRT-PCR validate analysis.

Gene	Primer sequences (5'-3')
GAPDH	F: GTGCCAAGAAGGTTATCATCTCTG<br>R: CTCATGCTCATTAACACCAACAAC
UR1	F: GCCATGGAAGTTTCAGGTGT<br>R: ACCGGGCACTGCAGTATAAC
UR2	F: GGCTTTTTCTTCCTCCATCC<br>R: GGAGTTCGCCAAGTGTGAAT
UR3	F: CTTCATGAATGGCCTCTGGT<br>R: GCATACACTCTCGCTGATCG
UR4	F: TCCGTGCAGATGAAGAACTG<br>R: CCGCGTTATCCCTCAGTTTA
UR5	F: CGATGAGCACTGCCAAGTAA<br>R: GTCCCCCATTCACACAAAAC
UR6	F: ATCATCTCCCGCACATCTTC<br>R: AAACGGTTACGTGGAGTTCG
UR7	F: GAAGCAAGCTCTGGTGGTTC<br>R: CGATCCCTAATCTTCCACGA
UR8	F: AAAAGCTCCCTGTCCTCCTC<br>R: CCGCTAAAATCCATTCCAAC
UR9	F: CCATACCGTCGCGTTTTATT<br>R: AGTCTTCTTCTGCCCCCATT
UR10	F: AGCAACGAAAGCCAGAAAGA<br>R: TTTTAATGTCGGTGGGCTTC
DR1	F: ATCCCATCCCCTGGAATATC<br>R: TTCTCCTCCAAGTCCCTCCT
DR2	F: TCCAATGACCACAAAAACGA<br>R: TTCTTCTCCGCAGACCACTT
DR3	F: TGTCACCCCAAATCTCATCA<br>R: TCTGGTGATGTGGTGCAGTT
DR4	F: CCAGCCTCCATCTCTCACTC<br>R: GGCAGTATCGAAGGTTCCAA
DR5	F: CTCAAATGCAGAAGCCACAA<br>R: TATGCACCAGAGGTGACAGC
DR6	F: AGATTGTGAGCATTGCGTTG<br>R: TCCTATGGCATTAGCGGTTC

Supplementary Figures

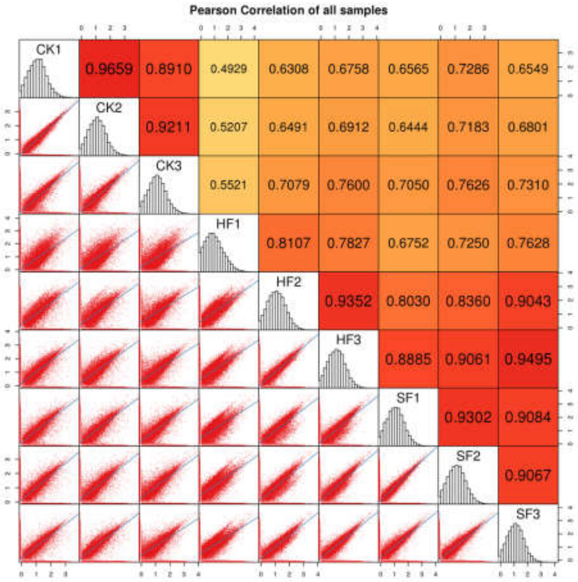

Figure S1. Pearson correlation of all samples.

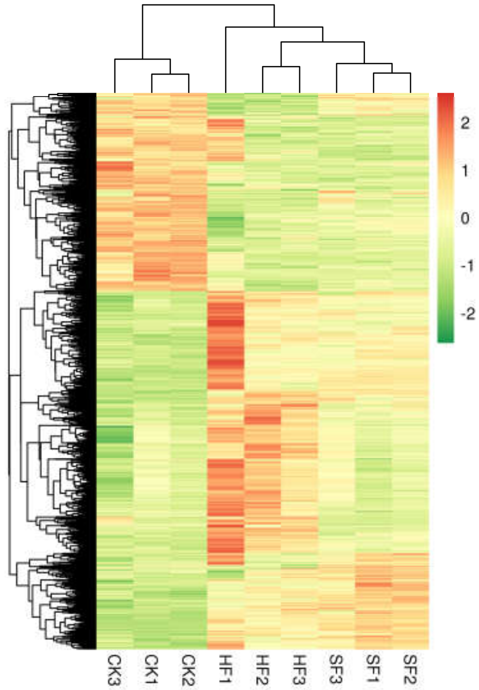

Figure S2. Heat map of gene expressions in the three libraries with three replicates.

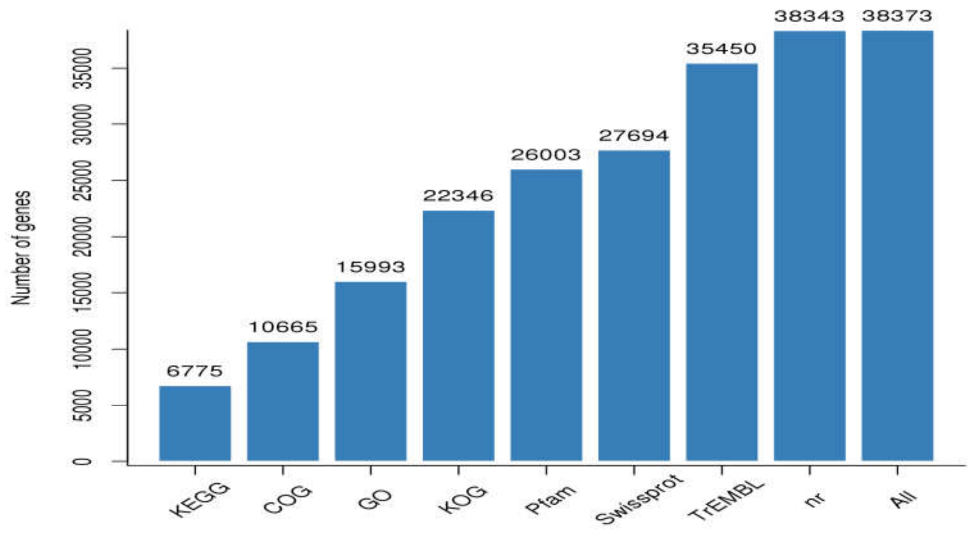

Figure S3. The number of the unigenes annotated in the databases.

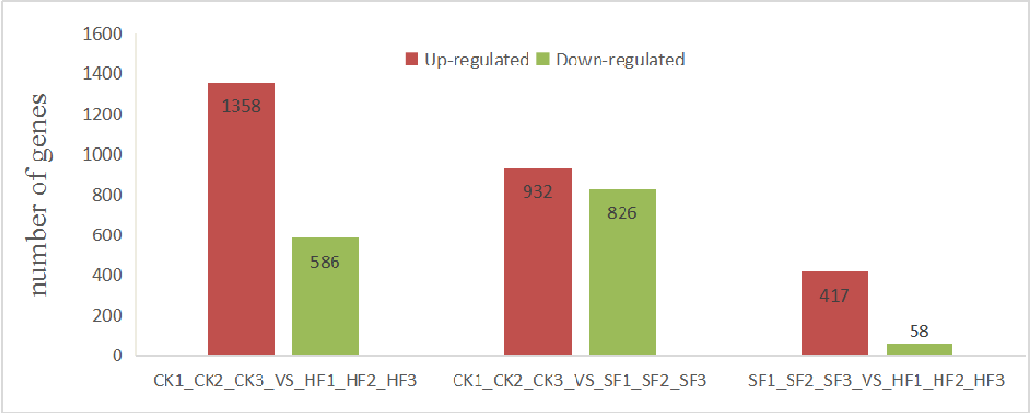

Figure S4. The number of the identified DEGs in the three libraries.

A

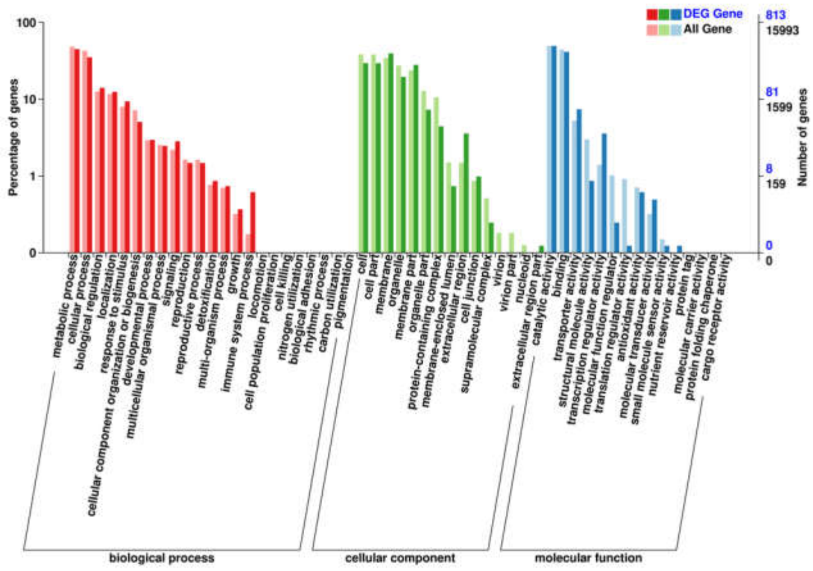

B

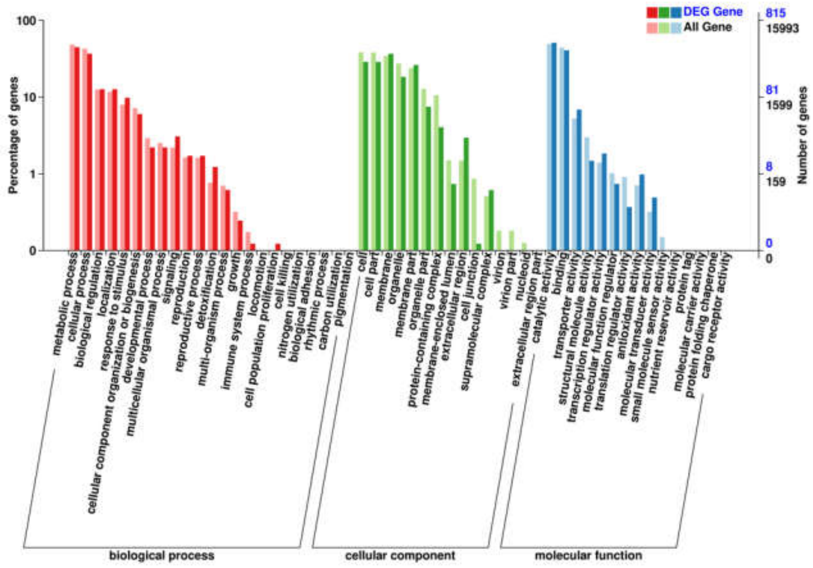

Figure S5. The GO terms classification of all gene and DEGS. (**A**) GO classification annotation in CK1_CK2_CK3_vs_SF1_SF2_SF3. (**B**) GO classification annotation in CK1_CK2_CK3_vs_HF1_HF2_HF3.
